# Synaptic effects of ethanol on striatal circuitry: therapeutic implications for dystonia

**DOI:** 10.1111/febs.16106

**Published:** 2021-07-16

**Authors:** Paola Imbriani, Giuseppe Sciamanna, Ilham El Atiallah, Silvia Cerri, Ellen J. Hess, Antonio Pisani

**Affiliations:** ^1^ Department of Systems Medicine University of Rome ‘Tor Vergata’ Italy; ^2^ IRCCS Fondazione Santa Lucia Rome Italy; ^3^ IRCCS Mondino Foundation Pavia Italy; ^4^ Departments of Pharmacology and Chemical Biology and Neurology Emory University Atlanta GA USA; ^5^ Department of Brain and Behavioral Sciences University of Pavia Italy

**Keywords:** alcohol, basal ganglia, corticostriatal plasticity, dystonia, myoclonus, striatum

## Abstract

Alcohol consumption affects motor behavior and motor control. Both acute and chronic alcohol abuse have been extensively investigated; however, the therapeutic efficacy of alcohol on some movement disorders, such as myoclonus‐dystonia or essential tremor, still does not have a plausible mechanistic explanation. Yet, there are surprisingly few systematic trials with known GABAergic drugs mimicking the effect of alcohol on neurotransmission. In this brief survey, we aim to summarize the effects of EtOH on striatal function, providing an overview of its cellular and synaptic actions in a ‘circuit‐centered’ view. In addition, we will review both experimental and clinical evidence, in the attempt to provide a plausible mechanistic explanation for alcohol‐responsive movement disorders, with particular emphasis on dystonia. Different hypotheses emerge, which may provide a rationale for the utilization of drugs that mimic alcohol effects, predicting potential drug repositioning.

## Graphical abstract

Alcohol EtOH improves clinical manifestations of different hyperkinetic movement disorders, including some dystonias. The underlying mechanisms of these effects remain partially unknown. We discuss how EtOH possesses a specific ability to “normalize” pathophysiologic changes in the entire basal ganglia circuit, a unifying pathophysiological feature in common with these disorders. Elucidating the mechanisms by which EtOH exerts its effects may help to identify new EtOH‐mimicking drugs without alcohol’s side effects.

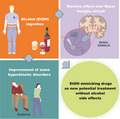

**Abbreviations**
ChIs: striatal cholinergic interneurons
D2R: dopamine D2 receptor
DBS: deep brain stimulation
DLL: long‐lasting disinhibition
DLS: dorsolateral striatum
DMS: dorsomedial striatum
eCBS: endocannabinoids
EPSCs: excitatory postsynaptic currents
EPSPs: excitatory postsynaptic potentials
FSIs: fast‐spiking interneurons
GHB: gamma‐hydroxybutyrate
GPi: globus pallidus pars interna
IPSCs: inhibitory postsynaptic currents
LTD: long‐term depression
LTF: long‐term facilitation
LTP: long‐term potentiation
LTSIs: low‐threshold spiking interneurons
MD: myoclonus‐dystonia
mGluRs: metabotropic glutamate receptors
MSNs: medium spiny neurons
NAc: nucleus accumbens
SGCE: epsilon‐sarcoglycan
SNc: substantia nigra pars compacta
SXB: sodium oxybate
VIM: thalamic ventral intermediate nucleus
VTA: ventral tegmental area

## Introduction

Alcohol acute administration leads to a variety of events, from initial anxiolytic and euphoric effects to severe intoxication with impairments in cognitive and motor performance, depending on the extent of intake. In contrast to other drugs of abuse, with circumscribed molecular targets (such as opiates), EtOH is a nonspecific drug: it acts on several molecular targets at neuronal and synaptic level in different brain areas. This is easily understandable, considering its ubiquitous distribution in the body and brain within minutes of intake. Indeed, previous studies have reported that alcohol interferes with GABAergic neurotransmission, but also other neurotransmitters, such as serotonin, dopamine, glutamate, cannabinoid, and beta‐endorphin, are targets of even low doses of EtOH [1], thus affecting both excitatory and inhibitory synaptic transmission. Further molecular targets include ligand‐ and voltage‐gated channels as well as a variety of synaptic proteins [2].

Empirical evidence shows that EtOH possesses a peculiar ability to improve the clinical manifestations of some hyperkinetic movement disorders; nevertheless, the complexity of its pharmacological characteristics poses significant difficulties to the interpretation of the mechanisms behind its effectiveness. Here, we will provide an overview of cellular and synaptic actions of EtOH in a ‘circuit‐centered’ view, focusing on basal ganglia function. Although the contribution of cerebellum in the effects of EtOH is well‐established, we will specifically focus on the striatum, the largest input station of the basal ganglia, that is critically involved in motor control and motor learning, decision‐making, and reward processing [3], functions that are all impaired during EtOH intoxication. We will analyze the potential role of striatal circuits in EtOH responsiveness, evaluating the rationale for potential clinical trials and drug repositioning for movement disorders, focusing on dystonia. This brief review does not pretend to provide an exhaustive summary of the complex molecular effects behind EtOH mechanisms of action, for which the reader is referred to other recent excellent references [2, 4, 5, 6].

## Effects of ethanol on striatal neuron excitability

The effects of EtOH are mediated by low‐affinity interactions with multiple molecular targets, including GABA_A‐B_ receptors, nicotinic acetylcholine receptors, ionotropic glutamate receptors, and glycine receptors [2]. Despite the effects of EtOH on specific molecules in different brain areas, the current trend in alcohol research abandons the ‘single‐target’ view of EtOH’s actions and instead examines its effects in a ‘circuit‐centered’ mode, which can be useful to better comprehend its overall clinical effects.

**Fig. 1:**
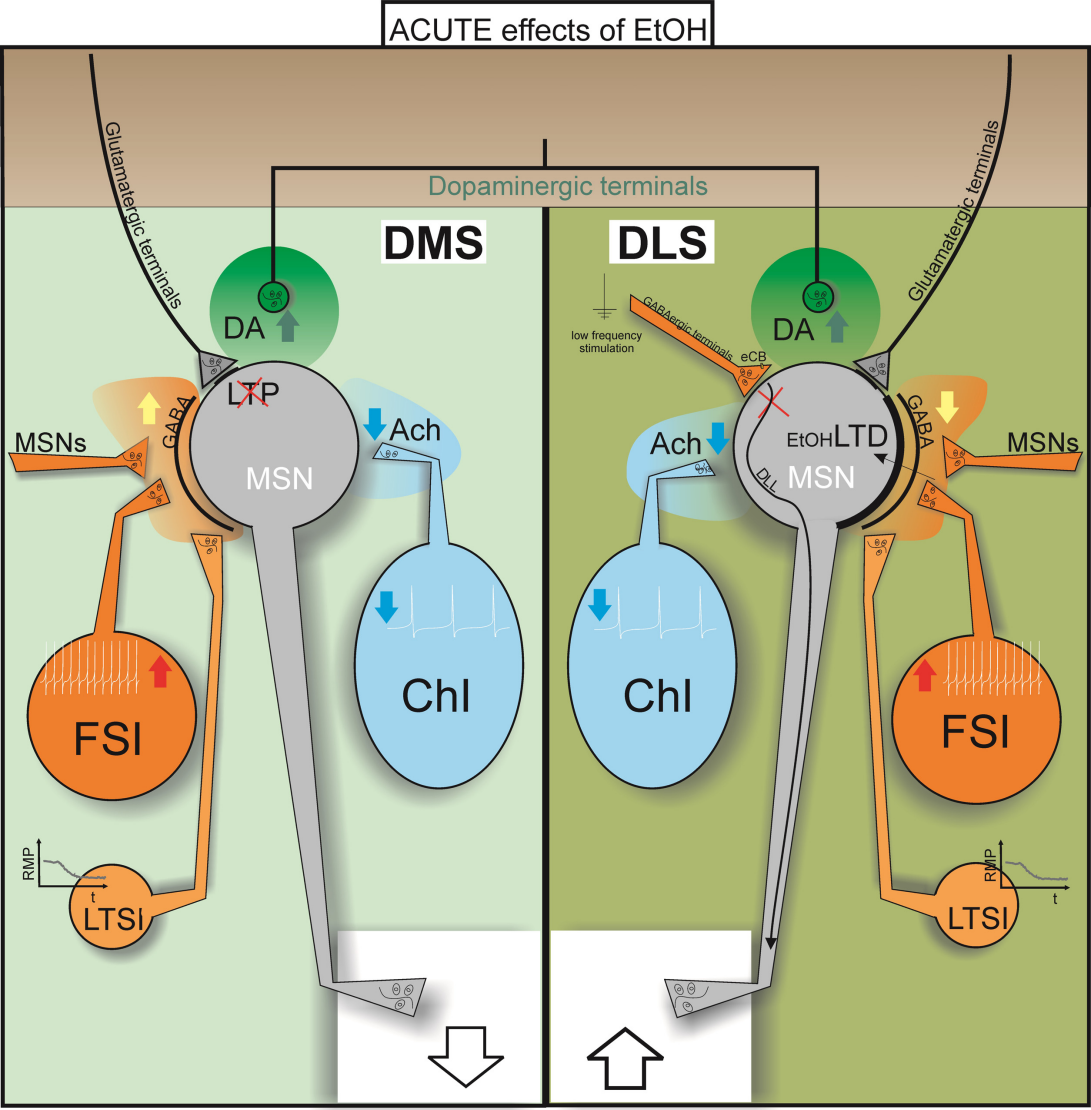
Effects of acute ethanol exposure on the dorsal striatum. The Figure summarizes the acute effects of EtOH on specific striatal neuronal subtypes and on synaptic transmission and plasticity. Acute EtOH has inhibitory effects on LTSIs, inducing membrane hyperpolarization, and on ChIs, decreasing their firing activity and, consequently, the release of ACh (light blue area, blue down arrow). The reduced local cholinergic tone leads to MSN hyperpolarization. Instead, acute EtOH induces membrane depolarization of FSIs followed by spontaneous bursts of action potentials (red up arrow). Low EtOH doses enhance firing of dopaminergic neurons, leading to increase in extracellular DA (green area, green up arrow). In the DLS, acute application of EtOH depresses the inhibitory MSN‐MSN and FSI‐MSN synapses (orange area, yellow down arrow) with a long‐term effect, which is considered a form of EtOH‐LTD. The net effect is a disinhibition of MSNs output from the dorsolateral striatum (big up arrow). At GABAergic synapses, EtOH prevents eCB‐mediated DLL of striatal output and reduces LTD induced by low‐frequency stimulation. In the DMS, acute EtOH potentiates GABAergic activity (orange area, yellow up arrow). At glutamatergic synapses, LTP is inhibited by acute application of EtOH (10 mm). The net effect is an inhibition of MSNs output from the dorsomedial striatum (big down arrow). DLS, dorsolateral striatum; DMS, dorsomedial striatum; FSI, fast‐spiking interneuron; MSN, medium spiny neuron; LTSI, low‐threshold spiking interneuron; ChI, cholinergic interneuron; DA, dopamine; Ach, acetylcholine; LTD, long‐term depression; LTP, long‐term potentiation; eCB, endocannabinoid; DLL, long‐lasting disinhibition; RMP, resting membrane potential.

Pioneering *in vitro* studies, based on the use of brain slice preparations, evaluated the acute effects of EtOH highlighting its high specificity in terms of brain areas and neuronal types, which reflects interactions with different receptors and ion channel subunits, thus justifying the effects on intrinsic excitability [7, 8, 9], but also on synaptic transmission and plasticity [10], [11]. Accumulating experimental evidence demonstrates that EtOH can affect striatal circuitry and the flow of information through the basal ganglia to the cortex by acutely affecting striatal projection neuron excitability and plasticity, as well as responses of specific striatal neuronal subtypes (Fig. 1).

In 2011, Blomeley *et al*. performed an elegant electrophysiological study on different interneuron subtypes in the dorsolateral striatum (i.e., fast‐spiking interneurons, low‐threshold spiking interneurons, cholinergic interneurons), describing how they are modulated by acute exposure to EtOH and how their responses may affect projection neurons [12]. The influence of EtOH on the excitability of striatal GABAergic interneurons can be summarized as follows: low‐threshold spiking interneurons (LTSIs) show acute ethanol‐induced hyperpolarization, while fast‐spiking interneurons (FSIs) exhibit a significant EtOH‐induced membrane depolarization (due to suppression of potassium current) followed by spontaneous bursts of action potentials. EtOH also has a strong inhibitory effect on striatal cholinergic interneurons (ChIs) spontaneous activity, as their firing frequency is decreased by potentiation of calcium‐activated potassium currents. The cell type‐specific effects of EtOH (i.e., inhibition of ChIs and LTSIs and increase of excitability of FSIs) depend on its specific interactions with ion channels of distinct type/subunit composition that are differentially expressed by these interneurons. In medium spiny neurons (MSNs), the large majority of the striatal neuronal population, acute bath application of EtOH causes hyperpolarization accompanied by a decrease in input resistance; however, when EtOH is applied in the presence of TTX (to synaptically isolate MSNs from neighboring neurons), no significant effects were recorded, suggesting an indirect action on MSNs, mediated by surrounding striatal interneurons, and mainly by ChIs. This is not surprising since the decrease in local cholinergic tone leads to MSN hyperpolarization due to reduced tonic activation of postsynaptic M1 muscarinic receptors through a mechanism involving Kir2 channels [12, 13]. These effects are accompanied by a downregulation of both glutamate and GABA ionotropic receptors in MSNs (as demonstrated by decreased evoked EPSCs and IPSCs amplitude, respectively). Hence, in this *ex vivo* slice preparation, striatal MSNs become less responsive to both excitatory and inhibitory synaptic stimuli after acute treatment. It should be noted that this response is restricted to striatopallidal MSNs, as striatonigral projection neurons are not depolarized by M1 receptor activation [13].

EtOH also acts on midbrain dopamine neurons, leading to well‐known reinforcing effects. Indeed, acute EtOH exposure stimulates the firing activity of midbrain neurons of the ventral tegmental area (VTA), leading to an increase in extracellular dopamine concentrations within the VTA [14]. The increase in dopamine neuron firing is also associated with increased dopamine concentrations in the nucleus accumbens (NAc), as evidenced by both experimental and clinical studies [4, 15]. EtOH also enhances firing of substantia nigra pars compacta (SNc) dopaminergic neurons, which underlies the increase in extracellular dopamine observed in the striatum after EtOH exposure *in vivo* [5]. However, at higher doses, EtOH decreases the evoked release of dopamine from terminals, probably acting through nicotine acetylcholine receptors (nAChR) in the NAc core, although this may not be the only mechanism involved [16, 17]. The same effect has been reported in the caudate‐putamen of adult male rats, where bath application of EtOH inhibits dopamine release, but only at high doses [18]. Thus, these apparently conflicting effects on striatal dopamine are indeed dose‐dependent: Low EtOH doses increase dopamine, whereas high concentrations dampen dopamine release. Moreover, the stimulation of GABA_A_ receptors on GABAergic interneurons in the VTA by lower doses of EtOH disinhibits dopaminergic neuronal activity, increasing their firing rate. GABA_A_ receptors can be found also on dopaminergic cells in the VTA, but GABAergic interneurons in the VTA are more sensitive to GABA_A_ agonists (and EtOH, too) than dopaminergic cells [19]. This would at least in part explain the dose‐dependent effects of EtOH on dopamine release, though further work is needed to delineate the main actors involved in these events.

## Effects on striatal synaptic transmission and plasticity

EtOH affects numerous aspects of synaptic transmission and neuronal connectivity, by affecting both excitatory and inhibitory neurotransmission, as well as the release of different neuromodulators, as mentioned above.

EtOH‐dependent potentiation of GABA_A_ receptors has been extensively investigated. Acute EtOH exposure induces facilitation of GABA transmission and increases synaptic inhibition, contributing to sedation and other aspects of intoxication. An increase of ‘tonic’ GABA_A_ currents has been demonstrated in several brain regions [20, 21]. The complex mechanisms underlying the facilitation of GABA transmission include potentiation of GABA release at the presynaptic level [22], enhancement of interneuron firing [23], and enhanced postsynaptic responses, in terms of increase in both amplitude and duration of GABA_A_‐mediated inhibitory postsynaptic currents (IPSCs) [24, 25]. EtOH potentiates the function of α/β/γ‐subunit‐containing receptors and of those containing α4 or α6 along with β and δ subunits [26, 27]. A critical role for the δ subunit in conferring enhanced sensitivity to alcohol on GABA_A_ receptors has been identified [28]. Moreover, recent studies suggest that EtOH potentiation of GABA_A_ receptor function depends on the phosphorylation of a serine residue on the γ2 subunit by protein kinase C (PKC) [2, 4, 29].

Additionally, acute EtOH inhibits all glutamate receptors in different brain areas. The most prominent action of EtOH is exerted on ionotropic NMDA receptors, which has been hypothesized to contribute to the cognitive impairment produced by EtOH [2]. The NR2B subunit of the NMDA receptor, in particular, is highly regulated by EtOH [10]. The effects of EtOH on glutamate release are controversial, with data reporting presynaptic potentiation and others reporting inhibitory effects [4].

EtOH can also modulate long‐term synaptic plasticity. Two major forms of synaptic plasticity, that is, long‐term depression (LTD) and long‐term potentiation (LTP), represent the synaptic processes contributing to memory and learning. It has been demonstrated that, in the hippocampus, EtOH inhibits LTP, through an inhibitory action on NMDARs [30], and enhances LTD, acting on NMDARs and mGluR type 5 (mGluR5) [31]. Alcohol modulation of long‐term synaptic plasticity has also been investigated in the striatum [4, 5, 32, 33, 34], where it leads to disruption of synaptic plasticity. Different electrophysiological studies have been conducted both in the sensorimotor (dorsolateral, DLS) striatum and the associative (dorsomedial, DMS) striatum, evaluating the effects exerted by acute and chronic EtOH exposure, as reported below and summarized in Table 1.

**Table 1:** Summary of acute and chronic effects of EtOH on synaptic transmission and plasticity in the dorsal striatum: evidence from electrophysiological studies.

Ethanol (EtOH) and synaptic transmission and plasticity in the dorsal striatum	
DLS	DMS
Acute EtOH ↑	Acute EtOH ↓
‐ Depression of GABAergic transmission [35] ‐ Induction of FSI‐MSN and MSN‐MSN EtOH‐LTD [37] ‐ No modulation of HFS‐LTD at glutamatergic synapses [39] ‐ Lack of eCB‐DLL at GABAergic synapses [39] ‐ Decreased LFS‐LTD at GABAergic synapses [39]	‐ Potentiation of GABAergic transmission [35] ‐ Dose‐dependent effect: EtOH [10mM] blocks HFS‐LTP, EtOH [50 mM] promotes HFS‐LTD at glutamatergic synapses [43] ‐ Induction of NMDA‐LTF [44, 45] ‐ Facilitation of HFS‐LTP at glutamatergic synapses [46]
Chronic EtOH ↑	Chronic EtOH ↑
‐ Depression of GABAergic transmission [35] ‐ Lack of eCB‐DLL at GABAergic synapses [39] ‐ Lack of HFS‐LTD at glutamatergic synapses [39, 41, 42] ‐ Decreased fEPSPs [36]	‐ Depression of GABAergic transmission [35] ‐ Induction of NMDA‐LTF [45] ‐ Facilitation of HFS‐LTP at glutamatergic synapses [46] ‐ MSN‐D1 excitation and MSN‐D2 inhibition [47, 48] ‐ Increased fEPSPs [36]

EtOH, ethanol; DLS, dorsolateral striatum; DMS, dorsomedial striatum; FSI, fast‐spiking interneuron; MSN, medium spiny neuron; HFS, high‐frequency stimulation; LTD, long‐term depression; eCB‐DLL, endocannabinoids‐mediated long‐lasting disinhibition; LFS, low‐frequency stimulation; LTP, long‐term potentiation; LTF, long‐term facilitation; fEPSPs, field excitatory postsynaptic potentials; MSN‐D1, MSNs of the direct pathway; MSN‐D2, MSNs of the indirect pathway. Up arrow indicates enhanced striatal output, and down arrow indicates decreased striatal output.

### Dorsolateral striatum

Acute application of EtOH to striatal slices has opposite effects on the two regions of the dorsal striatum: in the DLS, it inhibits GABAergic activity by reducing miniature inhibitory postsynaptic currents (mIPSC) frequency. Interestingly, acute EtOH exerts different effects on mice with a previous history of alcohol intake, compared to those exposed for the first time: in fact, acute EtOH no longer inhibits mIPSC frequency in EtOH drinking mice [35]. Also, long‐term voluntary EtOH consumption exerts opposing effects on synaptic transmission in the two striatal subregions: in the DLS, it induces a depression of field excitatory postsynaptic potentials (fEPSPs) amplitude [36]. In a recent study, Patton and Colleagues demonstrated that, in the DLS, acute EtOH depresses the inhibitory MSN‐ and FSI‐MSN synapses, and the effect persisted for 15 min following the end of EtOH application, suggesting to consider it a form of EtOH‐LTD. The FSI‐MSN EtOH‐LTD occurs through activation of presynaptic delta‐opioid receptor and, together with EtOH‐LTD at MSN‐MSN synapses, may contribute to the increased DLS output following EtOH exposure [37].

It is well‐established that striatal LTD is mediated by endocannabinoids (eCBs) by retrograde trans‐synaptic signaling at excitatory and/or inhibitory synapses [38]. Of interest, exposure to EtOH did not affect eCB‐mediated corticostriatal LTD [39]. However, at GABAergic synapses it prevented eCB‐mediated long‐lasting disinhibition (DLL) of striatal output and reduced LTD induced by low/moderate frequency stimulation, by modulating eCB‐signaling at presynaptic level. Thus, EtOH modulates eCB‐mediated striatal plasticity in a synapse‐specific manner [39].

**Fig. 2:**
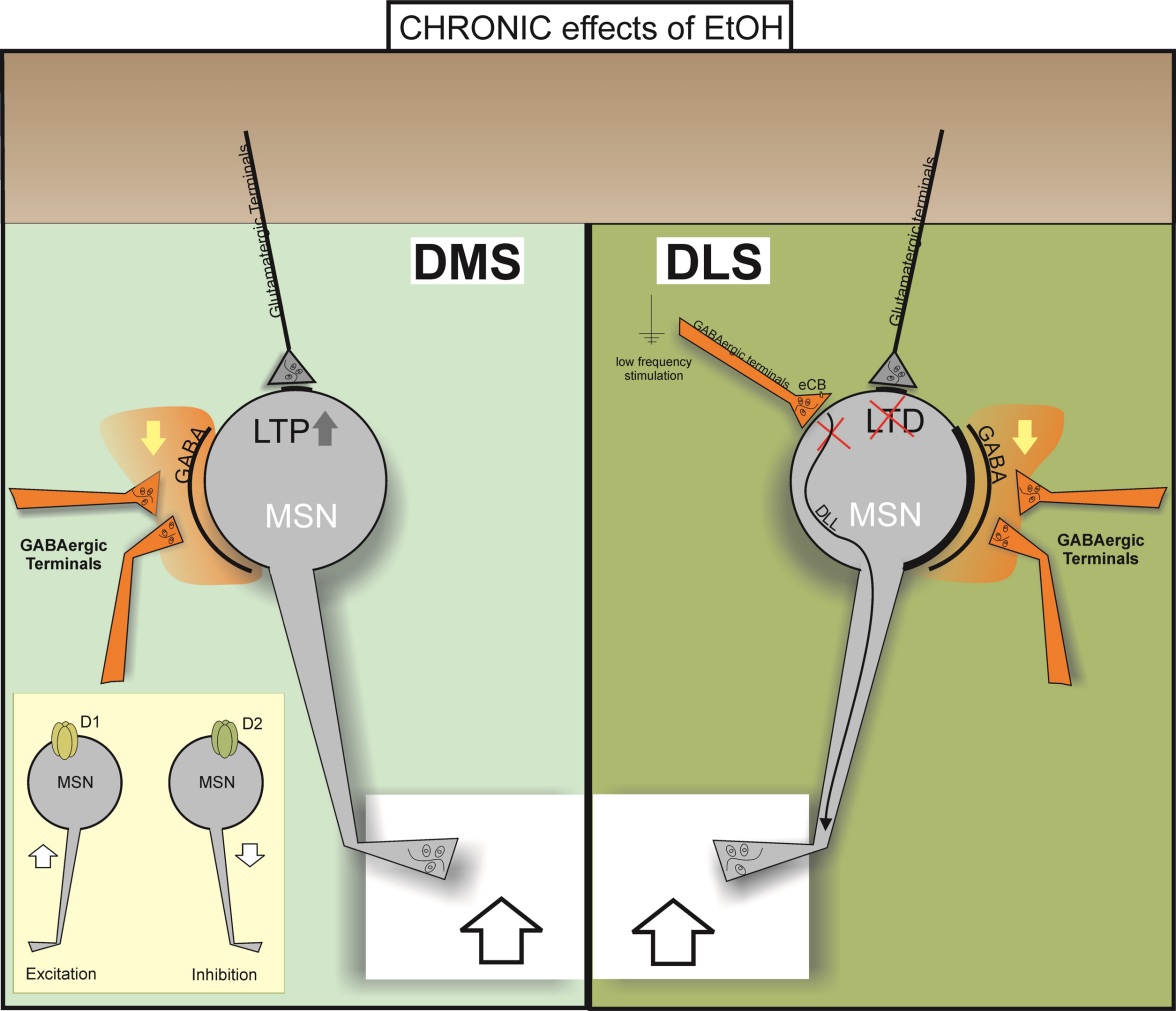
Effects of chronic ethanol exposure on the dorsal striatum. Chronic effects of EtOH on synaptic transmission and plasticity. GABAergic transmission is depressed in both DLS and DMS (orange area, yellow down arrow). In striatal slices from EtOH‐consuming rats, eCB‐DLL is impaired at inhibitory synapses and LTD is altered at excitatory synapses in the DLS. In the DMS, the repeated systemic administration of alcohol causes facilitation of LTP at glutamatergic synapses. Moreover, repeated cycles of excessive EtOH consumption and withdrawal potentiate glutamatergic transmission (excitation) in MSNs of the direct pathway (expressing dopamine D1 receptor) and potentiate GABAergic transmission (inhibition) in MSNs of the indirect pathway (expressing dopamine D2 receptor). The net effect is an overall disinhibition that contributes to enhanced output from both DLS and DMS (big up arrows). DLS, dorsolateral striatum; DMS, dorsomedial striatum; MSN, medium spiny neuron; LTD, long‐term depression; LTP, long‐term potentiation; eCB, endocannabinoid; DLL, long‐lasting disinhibition.

Chronic alcohol intake also affects striatal eCB signaling. In striatal slices from EtOH‐consuming rats, eCB‐DLL is impaired [40]. Moreover, chronic EtOH intake alters striatal LTD at excitatory synapses [40, 41, 42] (Fig. 2).

### Dorsomedial striatum

As opposed to DLS, acute EtOH potentiates GABAergic activity in the DMS by increasing mIPSC frequency; moreover, a history of EtOH drinking alters the acute alcohol effects on GABAergic transmission, inducing a decrease of mIPSC frequency rather than an increase [35].

Long‐term voluntary EtOH consumption results in an increase of fEPSPs amplitude; while the decrease in evoked potentials observed in the DLS is short‐lasting, the enhanced excitability in the DMS is not restored by abstinence, suggesting a long‐lasting effect [36].

A previous study on the effects of acute EtOH on long‐term synaptic plasticity at MSNs in the DMS showed that EtOH dose‐dependently alters the direction of striatal synaptic plasticity: at 10 mm EtOH, NMDAR‐dependent LTP is completely abolished, while higher concentration (50 mm) promotes LTD. The authors hypothesized that this action cannot be explained by the inhibition of NMDARs alone, but additional targets might be involved (i.e., potentiation of D2‐mediated signaling, activation of CB1 receptors) [43].

Acute treatment with EtOH increased tyrosine phosphorylation of the NR2B subunit of the NMDAR, resulting in a decrease in NMDAR‐mediated EPSCs; after EtOH washout, the NMDAR EPSCs gradually recovered and then increased above baseline for > 30 min. This long‐term enhancement of NMDAR activity after washout was attributable to a postsynaptic mechanism and named long‐term facilitation (LTF) [44, 45]. The same authors later demonstrated that acute exposure of striatal slices to EtOH facilitates the induction of LTP at glutamatergic striatal synapses and that the repeated systemic administration of alcohol causes an NR2B‐NMDAR‐dependent facilitation of LTP, through a long‐lasting increase expression of the GluR1 and GluR2 subunits of AMPARs at membrane level [46]. Since, as previously reported, Yin *et al*. [43] showed that LTP in the DMS is inhibited in the presence of EtOH, the authors deduce that this difference may be ascribable to the distinct time points at which LTP is induced: When the HFS protocol is delivered in the presence of EtOH, LTP is inhibited, but when it is delivered after EtOH withdrawal, LTP is facilitated. These mechanisms might underlie aberrant synaptic plasticity of the DMS, as this effect was not observed in the DLS nor in the nucleus accumbens [45].

A more recent study focused on DMS showed that repeated cycles of excessive EtOH consumption and withdrawal induce changes in synaptic strength by selectively potentiating glutamatergic transmission in MSNs of the direct pathway (expressing dopamine D1 receptor) and GABAergic transmission in MSNs of the indirect pathway (expressing dopamine D2 receptor). This excitation of D1‐MSNs and inhibition of D2‐MSNs may control alcohol‐associated behaviors and reinforce alcohol consumption [47]. These results are in line with the observation that chronic alcohol consumption produces a long‐lasting increase in synaptic AMPAR function selectively in D1‐MSNs of the DMS [48]. Finally, in chronic intermittent binge‐like EtOH drinking mice, it was demonstrated that the glutamatergic transmission and the density of dendritic spines of MSNs are unchanged, while the GABAergic transmission is depressed in both DLS and DMS, suggesting an overall disinhibition that would likely contribute to enhanced output from the dorsal striatum [35] (Fig. 2).

Overall, these results suggest that EtOH consumption differently modulates neurotransmission in dorsal striatal subregions, causing an altered balance between DMS and DLS.

### Ventral striatum

EtOH also affects synaptic transmission and plasticity in the nucleus accumbens. Acute EtOH inhibits both LTP and LTD, probably acting through inhibition of NMDARs and of group I metabotropic glutamate receptors (mGluRs) as well as altered dopamine release [32]. Chronic EtOH exposure potentiates glutamatergic transmission and impairs LTD in D1‐positive MSNs, while D1‐negative MSNs show normal LTD [49, 50]. Thus, synaptic alterations in the NAc may contribute to behavioral adaptations to chronic EtOH.

Taken together, these studies show that the effects of EtOH exposure and washout on synaptic transmission and synaptic plasticity in the striatum are multifaceted and that alcohol can globally shape the striatal output: specifically, acute EtOH administration inhibits MSNs output from the associative striatum and disinhibits MSNs output from the sensorimotor striatum (Fig. 1, Table 1). The modulation of synaptic plasticity by alcohol in the dorsal striatum may be of relevance for understanding alcohol responsiveness in some movement disorders.

## Alcohol and movement disorders

Alcohol is known to exert an effect on motor symptoms in a variety of movement disorders. Essential tremor, myoclonus‐dystonia (MD), and other forms of dystonia are included in the spectrum of ethanol‐responsive movement disorders [6], although the exact mechanisms remain unclear. This situation is further complicated by the evidence that the ‘beneficial’ effects of alcohol are accompanied by well‐known adverse effects, by the tendency to cause rebound involuntary movements when it wears off and, consequently, by alcohol abuse [51]. In contrast, in other conditions such as paroxysmal nonkinesigenic dyskinesias, attacks are even triggered or exacerbated by acute alcohol exposure [52, 53], which adds a further level of complexity. Here, we will specifically discuss MD and mention other alcohol‐sensitive dystonias, as the effect of alcohol on essential tremor has been subject of recent surveys [6, 52, 54, 55].

## Myoclonus‐dystonia and alcohol responsiveness

### Clinical features and genetics

MD is a rare, inherited disease, with a prevalence of about 2 per 1.000.000 in Europe [56] and with onset usually occurring in childhood or early adolescence. It is characterized by a combination of myoclonic jerks and dystonia. Myoclonus is the predominant motor sign, consisting of brief ‘shock‐like’ jerks, usually affecting upper body and elicited by action, posture, and psychological stress. Mild to moderate dystonia is present in more than half of cases, mainly presenting as torticollis or writer’s cramp, but also as focal dystonia involving the lower extremities, cranial region, and larynx. Patients can also manifest postural tremor of the upper limbs [57, 58]. Psychiatric disorders have also been reported, including depression, anxiety, obsessive–compulsive disorder, personality disorders, alcohol abuse, and panic attacks [59, 60]. Early reports on alcohol responsiveness in MD [61] were later confirmed by the demonstration of significant improvement of motor symptoms in MD patients after alcohol intake [6, 62, 63]. The condition is genetically heterogeneous. In most cases, it is related to epsilon‐sarcoglycan (*SGCE*) gene mutations with autosomal dominant inheritance and reduced penetrance [64], configuring the SGCE‐related‐myoclonus‐dystonia (or DYT‐*SGCE*). Epsilon‐sarcoglycan is a transmembrane glycoprotein widely expressed in human tissues including the brain, where, nevertheless, little is known about its role. In the mouse brain, high expression levels of epsilon‐sarcoglycan mRNA have been found in the olfactory bulb mitral cell layer, cerebellar Purkinje cells, and neurons of the substantia nigra, ventral tegmental area, dorsal raphe nucleus, and locus coeruleus [65]. Intriguingly, the two major SGCE isoforms were found to be, respectively, enriched in post‐ and presynaptic membrane fractions, which suggests their possible roles in synaptic function of the central nervous system [66]. Over the years, novel genes causative of MD phenotypes have been reported, including the *KCTD17* gene, with high expression in the putamen, which is likely to be involved in postsynaptic dopaminergic transmission [67]; *CACNA1B* gene, with N‐type calcium channel activity, is thought to be crucial in controlling neurotransmitter release [68]; *RELN* gene encodes reelin, an extracellular matrix glycoprotein which seems closely involved in modulation of synaptic function in adulthood [69]. Actually, patients with mutations in genes other than *SGCE* may display different clinical phenotypes compared to ‘typical’ *SGCE*‐related MD. For example, in *KCTD17*‐mutated patients, dystonia tends to predominate over myoclonus, the distribution of myoclonus is less pronounced in proximal upper extremities and neck, and, to date, there are no reports of improvement of myoclonus with alcohol intake [70]. Therefore, recently Roze and colleagues have suggested that the term ‘myoclonus‐dystonia’ should be limited to patients with a *SGCE*‐like phenotype, while the use of the term ‘myoclonic dystonia’ should be preferred for non‐*SGCE* patients; moreover, they proposed modified diagnostic criteria for the syndrome of MD, based on the *SGCE*‐like phenotype, also including alcohol responsiveness [58]. It should be noted that some clinical studies show heterogeneity of alcohol response both between and within families, and this response does not appear to be specific to the genetic etiology of MD [54]. Indeed, a positive effect of alcohol on motor symptoms has also been reported in patients with a myoclonus‐dystonia‐like syndrome caused by *CACNA1B* [71] and *RELN* [72] mutations. Alcohol misuse has been reported in phenotype descriptions of MD patients. It is postulated that the increased rate of alcohol abuse in this disease is secondary to the ameliorative effects of alcohol on the motor symptoms, as the result of uncontrolled self‐medication, rather than a phenotypic expression of the *SGCE* gene [59, 62]. The lack of treatments with similar efficacy also contributes to increase the risk of alcohol dependence. However, further work is needed to understand the relationship between alcohol dependence and MD.

### A circuit‐centered view

The pathophysiological mechanisms underlying motor symptoms in MD are still not clear, and a crucial debate is whether symptoms are caused by a primary dysfunction of the striato‐pallido‐thalamo‐cortical pathway or of the cerebello‐thalamo‐cortical pathway, with possible contribution of additional cortical dysfunction [58]. Neurophysiological evidence supports the existence of a subcortical generator underlying motor symptoms [73, 74], even though the peculiar phenotype characterized by the co‐existence of psychiatric disorders may suggest diffuse brain dysfunction [75]. The electrophysiological pattern of myoclonus, including electromyographic (EMG) bursts with a mean duration of 95 ms, the absence of cortical correlate preceding myoclonus on jerk‐locked back‐averaged EEG, and the absence of giant somatosensory evoked potentials, is in favor of its subcortical origin [76]. Moreover, noninvasive brain stimulation techniques, such as transcranial magnetic stimulation, have confirmed the absence of abnormalities of cortical function in *SGCE*‐MD: normal or higher motor cortical excitability, depending on the protocol used; normal or only subtly reduced intracortical inhibition (mediated by GABA_A_ interneurons) of the motor cortex [74, 76, 77, 78]. An imaging study of an isolated case of inherited MD also showed abnormal activation in subcortical structures, specifically within the thalamus and the dentate nucleus [79]. Any cortical abnormalities detected in neurophysiological and neuroimaging studies are thought to be the consequence of basal ganglia dysfunction rather than a primary cortical dysfunction [60].

Some evidence supports deficits of cerebellar networks underlying MD pathophysiology, suggesting that *SGCE* mutations cause Purkinje cell dysfunction with GABAergic deficits, which may be transiently compensated by alcohol administration [63, 80]. A potential role of the cerebellum in the pathophysiology of MD is also supported by neuroimaging [73, 81] and structural [82] studies, as well as by the evidence of high expression of *SGCE* gene in the cerebellum [80]. Moreover, a neurophysiological study demonstrated impaired saccadic adaptation in the DYT11 patients, further confirming cerebellar dysfunction [83].

Additionally, a role of the ventral intermediate nucleus (VIM) of the thalamus has been hypothesized as potentially involved in the generation of myoclonus. This is also supported by the evidence that deep brain stimulation (DBS) targeting the VIM is effective in *SGCE*‐MD [84, 85].

Nevertheless, there is also significant clinical and experimental evidence supporting the assumption that MD is primary due to dysfunction of the basal ganglia. In clinical studies, MD appears to be correlated to an alteration of MSN excitability with disruption in striato‐pallido‐thalamo‐cortical circuits [86, 87].

Studies conducted in parallel with DBS demonstrated that myoclonus severity is associated with abnormal neuronal activity in the internal globus pallidus (GPi) [88]; in line with this, DBS could be effective in reducing myoclonus by restoring the discharge pattern in the GPi and reducing the cortical overactivation [89]. The observation that basal ganglia DBS provides good benefit for symptoms represents a key piece of evidence of the central role of basal ganglia in MD [90, 91].

Moreover, in MD patients with pronounced dystonia, EMG‐EMG coherence analysis showed abnormal intermuscular 3 to 10 Hz drive, while EEG‐EMG coherence analysis showed no significant coherence in the 15 to 25 Hz band, which reflects an altered cortical drive caused by basal ganglia dysfunction leading to abnormal motor activation, in common with other dystonias [92].

Clinical observations are also supported by experimental data. Results from animal models of MD have demonstrated that Sgce deletion impacts striatal function. Sgce heterozygous knock‐out (KO) mice exhibit motor deficits, myoclonus, and abnormal nuclear envelopes in striatal MSNs, although striatum‐specific Sgce conditional KO mice exhibited only motor deficits, without evidence of abnormal nuclear envelopes or myoclonus [93]. Thus, the authors propose the development of therapies targeting the striatum to compensate for the loss of epsilon‐sarcoglycan function in *SGCE*‐MD patients. Moreover, in Sgce KO mice Zhang *et al*. reported alteration of striatal dopaminergic transmission, as evidenced by a significant decrease of striatal dopamine D2 receptor (D2R) and an increase of dopamine release after amphetamine injection [94], which confirmed the previous observation of higher levels of striatal dopamine and its metabolites [93]. Moreover, recent experimental evidence in a genetic mouse model of *SGCE* deficiency demonstrated impaired corticostriatal LTD, which can explain the circuit abnormalities thought to underlie MD. This neurophysiological deficit was completely reversed by blockade of the adenosine A2A receptors, which could in turn induce a potentiation of DR2, given the reciprocal A2AR/D2R interaction on MSNs [95].

The evidence that Sgce deletion might affect dopaminergic transmission is also supported by clinical evidence. Patients with MD exhibit reduced striatal D2R availability [96]. Despite clinical benefit, GPi‐DBS had no significant effects on D2R binding, but patients who did not undergo this procedure showed a decrease of D2R binding, suggesting that GPi‐DBS could exert a stabilizing effect on dopaminergic pathways [97].

### Mechanistic hypotheses on alcohol responsiveness in MD

Although the underlying pathophysiology of MD requires further investigation, the direct and considerable connections between the cerebellum and the basal ganglia indicate that MD should be considered as a network disorder, in which the dysfunction in one node influences the activity of others [98]. Consequently, the responsiveness to EtOH observed in MD and in other movement disorders raises the issue of whether these conditions share common anatomical networks, and indicates that EtOH may temporarily reverse the alterations of these neural circuits. At the system level, it could be hypothesized that alcohol acts on MD and on alcohol‐sensitive dystonia by modulating the output from the dorsal striatum and, in turn, motor control. At cellular level, multiple targets should be considered. Dopamine exerts a central role in striatal LTD induction, through D2Rs, and EtOH has been shown to modulate LTD in the dorsal striatum [43]. Thus, EtOH could exert its beneficial effects by acting, either directly or indirectly through the endocannabinoid system, on dopaminergic system. Alternatively, an attractive hypothesis is that the striatal modulation mediated by EtOH could be due to its effects on interneuron excitability and more specifically on the ability to down‐regulate the tonic firing frequency of ChIs [12]. The reduced excitability would lower the levels of striatal acetylcholine, which has a well‐established role in long‐term synaptic plasticity alterations observed in dystonia models [99, 100]. Further experimental work is required to clarify these mechanisms. Lastly, the cerebellar hypothesis should also be taken into account. A recent preclinical evidence demonstrated that adult mice with knockdown of Sgce in the cerebellum, but not in the basal ganglia, develop overt motor symptoms, including dystonia, which can be improved by administration of ethanol [101]. Moreover, very recently, Frucht and Riboldi proposed a model to explain the clinical improvement with low doses of EtOH in MD, supporting an abnormal activation of the Purkinje cells and dentate nucleus and a likely mechanism of action of EtOH to normalize abnormal cerebellar output in this disorder, paving the way for future speculation [102].

A better understanding of the mechanism by which alcohol ameliorates symptoms in MD, and in alcohol‐sensitive movement disorders in general, may help elucidating the pathophysiology of these conditions.

## Other alcohol‐sensitive dystonias

The effect of alcohol is not limited to patients with MD, since a wider range of subtypes of dystonia has been reported to have marked alcohol responsiveness.

One of the first reports of an alcohol‐sensitive purely dystonic syndrome was that of a patient presenting with a combination of generalized dystonia and myorrhythmic movements with onset in early‐adulthood and negative family history. Medical treatments (trihexyphenidyl and carbamazepine) were ineffective, but dramatic improvement occurred after alcohol intake [103].

Another recent paper described the case of a 29‐year‐old male patient with generalized dystonia, for whom some anti‐dystonic treatments were ineffective while alcohol induced a dramatic improvement [104].

Cases of alcohol‐responsive focal dystonia are reported more frequently than generalized dystonias. These include the following: five patients with spasmodic torticollis who benefitted from intravenous infusion of an EtOH solution [105]; spasmodic dysphonia in DYT4 dystonia [106]; a case of primary writer’s cramp that was unexpectedly improved by drinking a small amount of alcohol [107]; a 66‐year‐old woman with jerky cervical dystonia caused by a mutation in the GCH1 gene (the cause of dopa‐responsive dystonia) and with striking alcohol sensitivity [108]. Moreover, a survey in a large population of patients with laryngeal dystonia confirmed what was already observed in case reports, that is a marked improvement of voice symptoms after alcohol ingestion; this responsiveness was not attributed to the presence of voice tremor [109].

A recent study conducted on more than 1200 patients with isolated dystonia reported an improvement of motor symptoms after alcohol consumption in almost a third of them. Alcohol responsiveness was not related to age, sex, or severity of dystonia, but there was a significant association with an earlier age at onset, distribution of dystonic symptoms (i.e., multifocal/generalized forms had higher rates of alcohol responsiveness than segmental ones), subgroups of focal dystonia (i.e., cervical and laryngeal dystonia responded better than cranial and limb forms), presence of tremor and a positive family history (e.g., DYT‐*TUBB4A* and DYT‐*SGCE*). The latter suggests that an underlying genetic contribution may represent a predictor of alcohol responsiveness in patients with dystonia [110]. These results reinforce the concept of an independent effect of alcohol on dystonia and may pave the way for future research focused on the identification of further genes associated with alcohol‐responsive dystonia.

## New and old drugs for the treatment of myoclonus‐dystonia and other alcohol‐sensitive dystonia

By looking at pharmacological agents that exert, at least to some extent, a beneficial effect on MD, it would be simplistic to attribute EtOH efficacy solely to its GABAergic action: Indeed, benzodiazepines and other drugs acting via GABAergic pathway have proven only limited efficacy compared to EtOH, and, on the flip side, other agents with different mechanisms of action, like anticholinergics or zonisamide, have shown to significantly improve motor symptoms.

Though EtOH alleviates symptoms of MD, it is not viable as a long‐term treatment due to its pharmacokinetic and pharmacodynamics properties (i.e., adverse effects, rebound effect, tendency to abuse/misuse). However, as our understanding of the mechanisms underlying the efficacy of EtOH increases, medications that mimic the effects of EtOH have gradually emerged for the treatment of MD (Table 2).

**Table 2:** Potential drugs for myoclonus‐dystonia and other alcohol‐sensitive dystonia as reported by previous case reports and clinical studies.

Drug	Mechanism of action	Clinical effects	Adverse effects	References
**GABAergic agents**				
Benzodiazepines (clonazepam, diazepam)	GABA_A_ receptors agonists	++ in MD (mostly on myoclonus)	Sedation	[57, 60, 111, 112]
Zolpidem	alpha1 subunit‐containing GABA_A_ receptors agonist	+++ in MD (1 case)	Sedation	[113]
Sodium oxibate (SXB)	GABA_B_ and GHB receptors agonist	++ in MD (myoclonus) +++ in laryngeal dystonia	Sedation, dizziness, headache, respiratory depression, seizures, coma	[6, 102, 114, 117]
Gamma‐hydroxybutyrate (GHB)		+++ in MD (1 case)		
**Antiepileptics** Valproate	Sodium channels blocker, GABA agonist	+ in MD (myoclonus)	Nausea, diarrhea, hair loss, tremor, hepatotoxicity, drowsiness	[121, 125, 126]
Levetiracetam	Multiple actions (i.e., modulation of neurotransmitter release, calcium signaling)	++/− in MD	Psychiatric disturbances, toxic epidermal necrolysis	[127, 128]
Zonisamide	Sodium and low‐threshold T‐type calcium channel blocker, GABA agonist	++ in MD (both myoclonus and dystonia)	Headache, nausea, fatigue, sleepiness, diarrhea	[6, 123]
**Anticholinergics** (trihexyphenidyl, benztropine mesylate)		+++ in MD (mostly on dystonia)	Dry mouth, constipation, urinary retention, bowel obstruction, blurred vision	[60, 118, 119, 120, 121, 122]
**Levodopa**		++ in MD (myoclonus)	*//*	[129]
**Tetrabenazine**	VMAT2 inhibition	++ in MD (mostly on myoclonus)	Sedation, fatigue, insomnia, depression, anxiety, nausea	[130]

+, mild benefit; ++, modest benefit; +++, significant benefit; −, no benefit.

Benzodiazepines, that reduce neuronal excitability via GABAergic mechanisms, and other GABA modulators, like primidone and zolpidem, provide only mild or no improvement in patients with MD and produce adverse effects that lead to discontinuation [57, 60, 111, 112, 113].

A novel oral drug, developed as a GABAergic agent, and currently marketed for the treatment of cataplexy in narcoleptic patients, is sodium oxybate (SXB), the sodium salt of gamma‐hydroxybutyrate (GHB). SXB has been reported to be effective in MD, with a good tolerability and mild dose‐dependent sedation [114]. GHB, which is used in the treatment of alcohol withdrawal, was also reported effective in a patient with alcohol‐sensitive myoclonic jerks and dystonia [115]. The exact mechanism of SXB’s antimyoclonic activity remains unknown. GHB is supposed to have a low affinity to the metabotropic GABA_B_ receptor, either directly or via conversion to GABA [114] as well as an action via the GHB receptors that bind to extrasynaptic GABA_A_ receptors [116]. However, other mechanisms of action may be involved, since other GABA_B_ agonists, such as baclofen, have minimal effects on myoclonus [102]. A recent study on 531 patients with alcohol‐responsive laryngeal dystonia treated with SXB demonstrated an impressive improvement of dystonic symptoms, presumably as a result of the modulation of the abnormal plasticity within the cortical and subcortical circuitry [117]. It is worth noting that what emerges from clinical trials is that the response to small doses of alcohol in *SGCE*‐MD, adductor spasmodic dysphonia (ADSD), and abductor spasmodic dysphonia (ABSD) predicts the response to SXB [102].

Several studies confirmed good responses to anticholinergics in patients with MD who did not respond to other treatments (i.e., clonazepam and levodopa), even if their use can be accompanied by side effects [60, 118, 119, 120, 121, 122].

The efficacy of some antiepileptics in the treatment of MD patients has been described. Among these, zonisamide has been recently reported to be well‐tolerated and effective against action myoclonus and myoclonus‐related functional disability, in a way similar to the effects of alcohol, in a cohort of MD patients [123]. Thus, this treatment has been proposed as the first‐line option in mild to moderate patients and in forms not eligible for DBS [58, 124]. Valproate has been used to treat myoclonus, but with only modest benefit [121, 125, 126]. Levetiracetam has also been tried with variable results [127, 128]. Improvement with both levodopa [129] and tetrabenazine [130] has been described.

Beyond pharmacological therapies, in patients with severe and medical‐refractory forms of MD, DBS can be offered as a long‐lasting treatment. There are no controlled trials about DBS effectiveness in MD, but several reports can be found, using bilateral GPi, thalamic VIM, or a combination of them, as preferred targets [85, 87, 90, 91, 97]. Comparison between thalamic versus pallidal targets did not show a significant difference in efficacy of suppressing myoclonus, but GPi target improved dystonia to a greater extent than thalamic target [131]. So, DBS should be considered in selected cases of MD for its efficacy, both on myoclonus and dystonia, and its good tolerability [131].

## Conclusions

Different hyperkinetic movement disorders share the peculiarity of improving after alcohol ingestion. Nevertheless, the underlying mechanisms of these effects remain not completely known.

In this brief review, we propose that the improvement of motor symptoms with EtOH does not simply derive from an effect on GABA transmission. Instead, we propose that EtOH possesses a specific ability to ‘normalize’ the pathophysiologic changes in the entire basal ganglia circuit, a unifying pathophysiological feature in common with MD and other dystonias. A plausible model should explain why alcohol is able to improve the clinical manifestations of etiologically different disorders.

In this regard, a working hypothesis should be testable in animal models with different dystonia subtypes with the aim of exploring how EtOH modulates distinctive forms of striatal synaptic plasticity. Elucidating the mechanisms by which alcohol exerts its effects in MD and other movement disorders will contribute to our understanding of their pathophysiology. Indeed, techniques for cellular and circuit manipulation (DREADD, optogenetics) will provide a platform for elucidating the effects of EtOH on large‐scale brain circuitry and alcohol‐related behavior. Moreover, understanding the pathophysiology may reveal new therapeutic targets, lead to novel therapeutic approaches, and contribute to the rationale for potential clinical trials. Certainly, alcohol is not a viable option to treat movement disorders, due to its addictive potential and side effects, but exploiting drugs that mimic EtOH effects is a feasible alternative.

## Conflict of interest

The authors declare no conflict of interest.

## Author contributions

PI wrote the manuscript; AP, GS, EJH revised the manuscript; SC, GS prepared tables and figures.
